# LIPH promotes metastasis by enriching stem‐like cells in triple‐negative breast cancer

**DOI:** 10.1111/jcmm.15549

**Published:** 2020-07-02

**Authors:** Yixiao Zhang, Xudong Zhu, Xinbo Qiao, Xi Gu, Jinqi Xue, Yanshuo Han, Lisha Sun, Meizi Cui, Caigang Liu

**Affiliations:** ^1^ Cancer Stem Cell and Translational Medicine Laboratory Department of Breast Surgery Shengjing Hospital of China Medical University Shenyang China; ^2^ Department of Cadre Ward The First Hospital of Jilin University Changchun China

**Keywords:** epithelial‐mesenchymal transition, lipase member H, metastasis, prognosis marker, stem‐like cells, triple‐negative breast cancer

## Abstract

Lipase member H (LIPH), a novel member of the triglyceride lipase family. The clinical implications of its expression in breast cancer are still unclear. Therefore, in this study, we investigated the associations between LIPH and the tumorigenic behaviours of 144 triple‐negative breast cancer (TNBC) patients. The ratio and mammosphere‐forming ability of CD44+/CD24− stem‐like cells were tested. The role of LIPH in breast cancer cell migration and invasion was also evaluated. In addition, the effect of LIPH silencing on mitochondrial respiration was determined using the Seahorse assay. Finally, the effect of LIPH silencing on protein expression was determined via tandem mass tag‐based spectrometry and Western blotting. We found that LIPH expression was associated with metastasis in lymph nodes and distant organs (*P* = 0.025), resulting in poor survival among breast cancer patients (*P* = 0.027). LIPH knockdown significantly decreased both the ratio of CD44^+^/CD24^−^ stem‐like cells and their mammosphere‐forming ability. LIPH silencing promoted apoptosis, arrested cell cycle in the G2/M phase, mitigated the oxidation‐related oxygen consumption rate in the mitochondria, and reduced metabolism. LIPH inhibited adhesion between tumour cells and enhanced the epithelial‐mesenchymal transition. Tandem mass spectrometric analysis presented 68 proteins were differentially expressed in LIPH‐silenced cells and LIPH‐mediated modulation of tumour cell adhesion depended on integrin‐related CAPN2 and paxillin signalling. Overall, our findings provided strong evidence that LIPH up‐regulation promoted metastasis and the stemness of TNBC cells. Therefore, targeting LIPH is a potentially viable strategy for preventing metastasis in TNBC.

## 1 INTRODUCTION

Breast cancer is one of the most common malignant tumours in women and its incidence is increasing worldwide.^1^ Triple‐negative breast cancer (TNBC) is associated with an adverse prognosis and a relatively limited range of systemic therapeutic options.^2^ Moreover, basal‐like TNBCs are enriched in poorly differentiated breast cancer stem cells (BCSCs) with the CD44^+^/CD24^−^ phenotype^3^ and the frequency of CD44^+^/CD24^−^ BCSCs has been shown to be related to chemotherapy resistance of breast cancer.^4^, ^5^ Hence, an understanding of TNBC pathogenesis that leads to new therapeutic targets is urgently needed.

Lipase member H (LIPH, also known as mPA‐PLA1) is a member of the mammalian triglyceride lipase family localized on human chromosome 3q27‐q28 and is a novel member of the triglyceride lipase gene family.^6^ The encoded proteins hydrolyse triglycerides and phospholipids^7^ to generate fatty acids that facilitate intestinal absorption or serve as an energy source or energy reserve. Human LIPH shares 47% identity with phosphatidylserine‐specific phospholipase A1 (PS‐PLA1) and 46% with endothelial lipase (LIPG) and lipoprotein lipase (LPL).^8^ LIPH was also found to be correlated with hypotrichosis/woolly hair,^9^, ^10^ dyslipidaemia, and hypertensive disorders,^11^, ^12^ as well as lipid and energy metabolism disorders.^6^ Notably, recent studies have observed a correlation between LIPH and adenocarcinomas of the lung or oesophagus,^13^ indicating that LIPH might be involved in the metastasis of the cancers. We have previously shown that lipase H is differentially expressed in patients with breast cancer and is related to poor prognosis of breast cancer.^14^ However, the clinical implications and role of LIPH in promoting TNBC tumorigenesis is still unclear.

In this study, we have examined the impact of LIPH expression on the metastasis and prognosis of TNBC patients and the relationships between LIPH expression and the ratio of CD44^+^/CD24^−^ BCSCs. Furthermore, we investigated the potential mechanisms underlying the regulatory effect of LIPH on the key processes of tumour metastasis, such as cell‐cell adhesion, EMT, and mitochondrial metabolism. Our findings uncover new mechanisms by which LIPH regulates the stemness that enhances metastasis of TNBC and may, therefore, aid the therapeutic intervention of drug‐resistant TNBC.

## 2 MATERIALS AND METHODS

### 2.1 Patients and breast cancer specimens

A total of 144 TNBC specimens were obtained from patients, 5 were ductal carcinoma in situ (DCIS) and 139 were invasive ductal carcinoma (IDC) at China Medical University from March 2006 to October 2008. The inclusion criteria were as follows: (a) female patients aged 18‐70 years; (b) only unilateral invasive breast ductal carcinoma; (c) absence of distant metastasis.

Another 30 patients, treated for advanced breast cancer between January 2016 and December 2017 were also included. And biopsy specimens of primary and metastatic tumours (including liver, lung, and skin tumours) were subjected to immunohistochemical staining for LIPH.

The Ethnic Committee of China Medical University approved the experimental protocol and written informed consent was obtained from individual patients. The demographic and clinical data were recorded and patient follow‐up exams were conducted regularly. Breast cancer recurrence and cancer distant organ or positions’ metastasis were recorded through January 31, 2018.

### 2.2 Immunohistochemistry

The expression of LIPH in primary breast cancer tissue, including 5 cases of ductal carcinoma in situ (DCIS), 139 cases of invasive ductal carcinoma (IDC), as well as 30 pairs of primary/metastatic specimens, was characterized by immunohistochemistry as previously described.^14^ Briefly, tissue sections (4 µm) were dewaxed, rehydrated, and subjected to antigen retrieval. The sections were treated with 3% BSA and probed with an anti‐LIPH antibody (1:500, Proteintech Group, Inc, USA) overnight at 4°C. After washed, the sections were incubated with a secondary biotinylated antibody (Multilink Swine anti‐goat/ mouse/rabbit immunoglobulin; Dako Inc, USA), followed by Avidin‐Biotin Complex (1:1000 dilution, Vector Laboratories Ltd., UK). Specimens were then visualized using a 3,3’‐diaminobenzidine (DAB) kit (Fuzhou, China). LIPH expression levels were evaluated semi‐quantitatively and intensity levels of 0, 1, 2, and 3 corresponded to absent, weak, moderate, and strong expression, respectively, corresponding to <1%, 1%‐10%, 11%‐50%, and 51%‐100% positive cells, respectively. The signal intensity score was the sum of the signal intensity and positive cell percentage, where the maximum score was 6. Individual specimens with a score of ≤2 for anti‐LIPH staining were considered as LIPH‐negative.

### 2.3 Immunofluorescence

The CD44 and CD24 expression levels in breast cancer tissues were analysed by immunofluorescence. Briefly, tissue sections (4 µm) were dewaxed, rehydrated, and subjected to antigen retrieval, after which they were treated with 10% FBS and incubated with a mouse anti‐human CD44 monoclonal antibody (#3570; Cell Signaling Technology, Danvers, MA, USA) and a rabbit anti‐human CD24 monoclonal antibody (ab202073; Abcam, Cambridge, UK) at 4ºC overnight. After washing, the sections were incubated with the Alexa Fluor 647‐labelled goat‐anti‐mouse IgG and Alexa Fluor 488‐labelled goat‐anti‐rabbit IgG, followed by nuclear counterstaining with DAPI (C0060; Solarbio, Beijing, China). Fluorescent signals were observed under a fluorescent microscope and the percentages of CD44^+^/CD24^‐^ CSCs in 2000 total tumour cells from at least three sections were counted in a blinded manner.

### 2.4 Cells

Human breast cancer MDA‐MB‐231 cells were purchased from the American Type Culture Collection (ATCC, Manassas, VA, USA). They were cultured in Leibovitz's L15 medium (Thermo Fisher, Carlsbad, CA, USA) supplemented with 10% foetal bovine serum (FBS, Cellmax, Lanzhou, China) at 37ºC in a humidified atmosphere without CO_2_.

### 2.5 LIPH knockdown and transfection

MDA‐MB‐231 cells (4 × 10^5^ cells/well) were cultured in a 6‐well plate. When the cells approached 80% confluence, they were transfected with control shRNA (5’‐ TTCTCCGAACGTGTCACGT‐3’) or LIPH‐specific shRNA (5′‐cgAATGAAGTTAAGGTCCCTT‐3′, 45#; 5′‐ccAGGATTATAGGAATGGCAA‐3′, 46#; 5′‐gcCCACATATCTGGGTTTGTT‐3′, 47#) at a multiplicity of infection of 10. The cells were treated with 4 µg/mL of puromycin to generate LIPH stably silenced MDA‐MB‐231/NC and MDA‐MB‐231/shLIPH cells. The efficacy of LIPH silencing was verified via Western blot analysis.

### 2.6 Flow cytometric sorting and mammosphere formation

MDA‐MB‐231/NC and MDA‐MB‐231/shLIPH cells were blocked with anti‐CD16/CD32 and stained with FITC‐anti‐CD24 and PE‐anti‐CD44. Cells stained with isotype control, FITC‐anti‐CD24, or PE‐anti‐CD44 alone served as the controls. The percentages of CD44^−^/CD24^−^, CD44^−^/CD24^+^, CD44^+^/CD24^+^, and CD44^+^/CD24^−^ cells were analysed and the CD44^+^/CD24^−^ stem‐like cells were sorted via flow cytometry using a FACS Aria™ III cell sorter (BD Biosciences, San Jose, CA). The sorted CD44^+^/CD24^−^ cells were cultured in stem cell medium (SC, 10% human MammoCult™ Proliferation Supplements in MammoCult™ Basal Medium, Stem Cell Technologies, San Diego, CA, USA). The mammospheres formed were photographed using a phase‐contrast microscope and enumerated in a blinded manner.

### 2.7 Apoptosis assay

Apoptosis was evaluated using flow cytometry as a function of the proportion of phosphatidylserine‐exposing apoptotic cells via Annexin V–FITC and propidium iodide (PI) double staining. Cells were digested with no‐EDTA trypsin and washed with PBS. After adjusting the cell density to 2 × 10^5^ cells/mL, cells were resuspended in 195 μL of Binding Buffer and treated with 5 μL of Annexin V‐FITC and incubated at 25°C for 10 minutes in the dark. After sample washing with binding buffer, PI was added and incubated in the same manner, in accordance with the Life Technologies Apoptosis Assay protocol. A FACSAria III (BD Biosciences, USA) was used to measure the frequency of apoptotic cells. Data were analysed with the FlowJo software and dot plot graphs were used to represent viable (lower left quadrant), early‐phase apoptotic (lower right quadrant), late‐phase apoptotic or dead (upper right quadrant), and necrotic cells (upper left quadrant).

### 2.8 Cell cycle analysis

Cells were digested with trypsin, washed gently with PBS, resuspended in PBS at 1 × 10^6^ cells/mL, and centrifuged at 200 *g* for 5 minutes. The supernatant was discarded and the cell pellet was washed three times, treated with 70% precooled ethanol, and gently mixed. Samples were incubated at −20°C overnight and then centrifuged (300 *g* for 2 minutes) and washed with PBS. Thereafter, 200 μL of cycle reagent was added to each sample and incubated for 30 minutes in the dark. Samples were then analysed via flow cytometry.

### 2.9 Transwell assay

The effect of LIPH knockdown on breast cancer cell invasion and migration were determined via a transwell migration assay. Briefly, NC and shLIPH cells (2 × 10^4^ cells/well) were loaded in triplicate on the upper chamber of a 24‐well transwell plate in the situation of FBS‐free medium. The bottom chambers were complete medium of 10% FSB and these cells were cultured for 24 hours. The migrated MDA‐MB‐231 cells on the bottom surface of the membrane were stained with 0.1% crystal violet. The number of migrated MDA‐MB‐231 cells in five selected fields randomly were numbered using a phase‐contrast microscope and in a blinded manner.

### 2.10 Adhesion assay

Conventionally digested MDA‐MB‐231/NC and MDA‐MB‐231/shLIPH cells and/or MDA‐MB‐231/NC and MDA‐MB‐231/shLIPH cells treated with C67399 for 24 hours were prepared as cell suspensions (5 × 10^5^ cells/mL). The cells were then seeded to pre‐coated 96‐well plates (BesBio, Shanghai, China, BB‐48120) (100 µL/well), cultured for 1 hour, and washed twice with culture medium. Thereafter, 100 µL of culture medium containing 10% CCK‐8 was added to each well and the cells were incubated for 2 hours, after which the absorbance at 450 nm was determined using a microplate reader.

### 2.11 Mitochondrial respiration assay

The effect of LIPH silencing on mitochondrial respiration in MDA‐MB‐231 cells was determined using the Seahorse assay in an XF24 extracellular flux analyser (Seahorse Bioscience, North Billerica, MA, USA) in accordance with the manufacturer's instructions. Briefly, control and LIPH‐silenced MDA‐MB‐231 cells were cultured overnight in XF24 (V7) microplates (2 × 10^4^ cells/well). The cells were cultured in basic medium containing glucose, glutamine, and sodium pyruvate at 37°C in a non‐CO_2_ incubator for 1 hour and the basal oxygen consumption rates (OCR) of individual wells of cells were measured thrice at 5 minutes intervals. Subsequently, cells were sequentially treated with rotenone/antimycin A (Krebs cycle inhibitors, all from Sigma) to quantify ATP production, proton leak, maximal respiration, and spare respiratory capacity after each treatment using the equipped software, normalized to protein levels measured via the BCA assay.

### 2.12 Quantification of lysophosphatidic acid (LPA) levels via ELISA

LPA levels in culture supernatants from stably transduced cells or vehicle were quantified via sandwich ELISA (MM‐12905H1, MeimianBio, Wuhan, China) in accordance with the manufacturer's protocol.

### 2.13 Tandem mass spectrometry

The effects of LIPH silencing on protein expression in MDA‐MB‐231 cells were determined via tandem mass tag‐based spectrometry.^15^ Cells were transfected with shRNA and harvested at the 3rd or 4th generation and immediately transported in dry ice to Beijing Protein Innovation (Beijing) for further analysis. Briefly, sh LIPH and control NC cells were harvested and their proteins were extracted. By digestion using trypsin, these peptides were vacuum‐dried and labelled with TMT reagents according to the manufacturer's instructions (Thermo Fisher Scientific). The peptides from shLIPH and control NC cells were labelled in triplicate with TMT10 tags at room temperature for 3 hours and quenched with 5% hydroxylamine. The labelled samples were pooled, subjected to fractionation, and then analysed on an Orbitrap Q Exactive mass spectrometer (Thermo Fisher Scientific) coupled with HPLC via a nanoelectrospray ion source using an analytical column with a 5%‐45% gradient of 90% (v/v) CH_3_CN and 0.1% (v/v) formic acid run for 170 minutes with a flow rate of 250 nL/min. Data from the LC/MS analysis were screened using the Proteome Discoverer (Version 1.4) and SEQUEST HT software applications (Thermo Fisher Scientific).

The gene ontologies of differential proteins and genes were analysed for using the AMIGO (http://www.geneontology.org) and DAVID software applications and enrichments were evaluated using the Kyoto Encyclopedia of Genes and Genomes (KEGG, http://www.genome.jp/kegg) annotations. Differentially expressed proteins were further evaluated via cluster analysis and potential protein‐protein interactions were assessed.

### 2.14 Quantification of lysophosphatidic acid (LPA) levels via ELISA

LPA levels in culture supernatants from stably transduced cells or vehicle were quantified via sandwich ELISA (MM‐12905H1, MeimianBio, Wuhan, China) in accordance with the manufacturer's protocol.

### 2.15 Western blot analysis

Cell samples were lysed in NP40‐containing buffer with PMSF, protease inhibitors, and phosphatase inhibitors. Individual cell lysate samples (30 µg/lane) were separated via SDS‐PAGE on a 12% resolving gel and electro‐transferred to polyvinylidene difluoride membranes (Millipore, Hong Kong). The membranes were blocked with 5% non‐fat dry milk in TBST and incubated overnight at 4°C with primary antibodies, including anti‐human LIPH (1:500, Proteintech, Wuhan, China), anti‐GAPDH (Cell Signaling Technology, Danvers, MA), anti‐CD44 (60224‐1‐Ig), anti‐CD24 (10600‐1‐AP), anti‐Oct4 (11263‐1‐AP), anti‐Sox2 (11064‐1‐AP), anti‐vimentin (WL01960), anti‐MMP2 (WL01579a, WanleiBio, China), anti‐FAK p397 (ab24781, Abcam, UK), anti‐Integrin‐ß3 (WL02735b, WanleiBio), anti‐p‐AKT (WLp001, WanleiBio), anti‐AKT (WL0003b, WanleiBio), anti‐RAS (WL0257, WanleiBio), anti‐CAPN2 (abs137284), anti‐Paxillin (WL00415, WanleiBio), anti‐Vinculin (abs131199), and anti‐Tubulin‐α (2144s, CST, USA). The bound antibodies were detected with horseradish peroxidase (HRP)‐conjugated secondary antibodies (1:10 000). The immunoblotting signals were visualized using enhanced chemiluminescent reagents. The ratios of target proteins to control GAPDH were determined by densitometric analysis using the ImageJ software application (NIH, Bethesda, MD, USA).

### 2.16 Statistical analysis

Data are expressed as mean ± SD values. Differences among all groups were analysed using one‐way ANOVA, followed by the Newman‐Keuls test for post hoc analysis, while differences between two groups were analysed using the Student's t test. Disease‐free survival in each patient group was estimated using the Kaplan‐Meier method and the differences among groups were analysed using the Log‐rank test. All statistical analyses were performed using SPSS version17.0 (IBM, Armonk, NY, USA). A *P*‐value of <0.05 was considered statistically significant.

## 3 RESULTS

### 3.1 LIPH expression is related to metastasis and poor prognosis in TNBC patients

**FIGURE 1:**
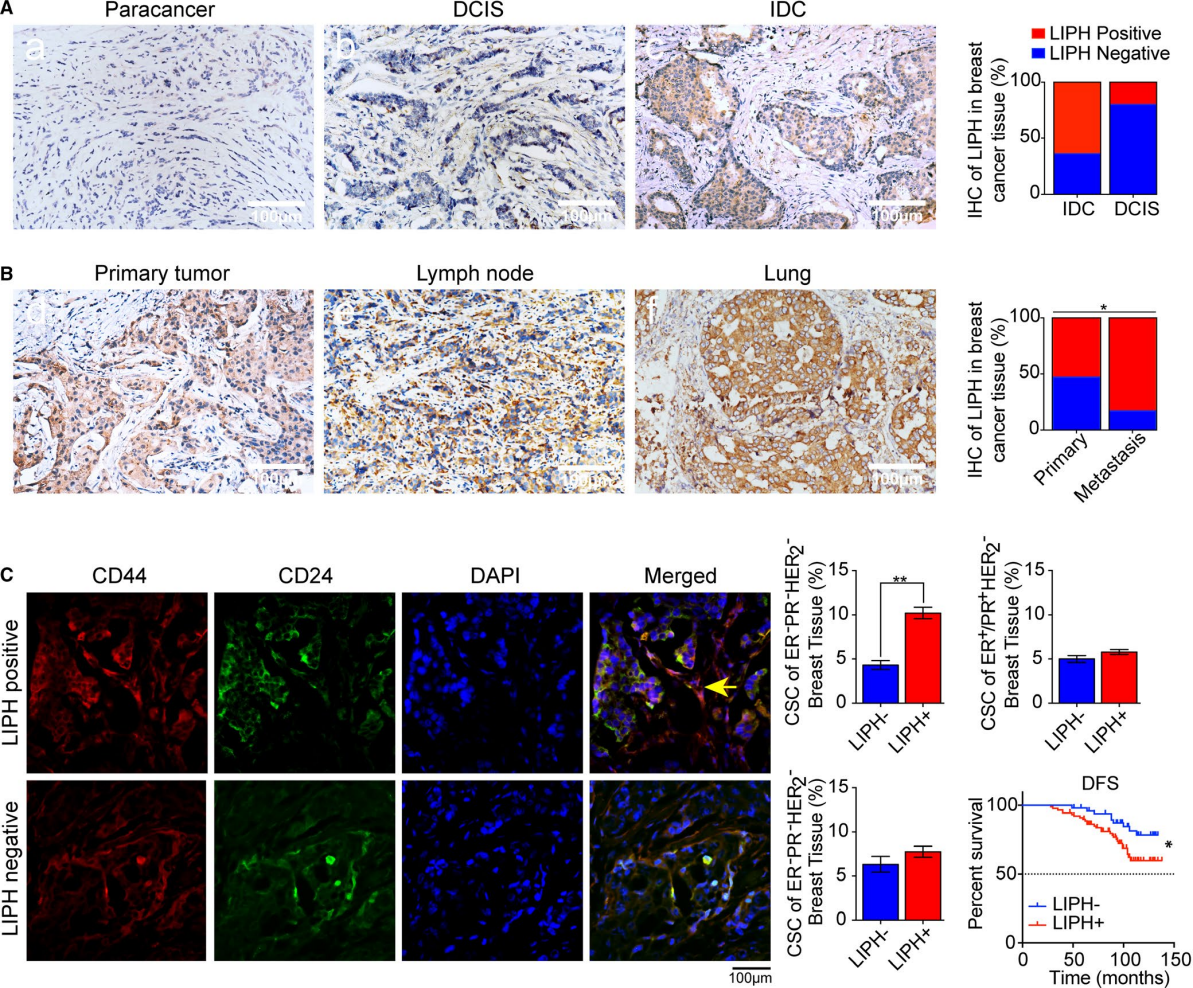
LIPH expression is associated with metastasis and poor prognosis in human TNBC. A, LIPH expression characterized by immunohistochemistry and stratified as high expressing in invasive ductal carcinoma (IDC) TNBC tumour tissues. A representative image of LIPH staining in paracancerous, ductal carcinoma in situ (DCIS) and IDC is shown. B, Representative LIPH immunohistochemical staining of lymph node metastasis and lung metastasis. LIPH is present in the alveolus pulmonis. Scale bar, 100 μm. C, Immunofluorescence of CD44^+^/CD24^−^ CSCs in LIPH + TNBC specimens was significantly higher than that in the corresponding LIPH‐ breast cancer. D, The association between LIPH expression and disease‐free survival (DFS)

LIPH was positively expressed in 89 out of 139 (64.03%) IDC specimens and predominantly expressed in the cytoplasm of tumour cells compared to weak expression of LIPH in 1 out of 5 (20%) DCIS specimens (*P* = 0.066, Figure 1A). To understand the role of LIPH in breast cancer metastasis, we further evaluated the expression of LIPH in 30 advanced breast cancer specimens with both primary and metastatic focus. LIPH showed significantly higher expression in metastatic samples than in primary tumours (*P* = 0.025, Figure 1B).

We further analysed LIPH expression and the frequency of CD44^+^/CD24^−^ cells in 139 specimens from patients with different molecular types of breast cancer by immunofluorescence (Figure 1C). After stratification of these specimens based on LIPH expression, we found that the percentage of CD44^+^/CD24^−^ CSCs in LIPH + TNBC specimens was significantly higher than the percentage in the corresponding LIPH‐ breast cancer (Figure 1C). Finally, LIPH‐positive breast cancer in patients with TNBC was associated with a significantly shorter disease‐free survival (DFS) compared to LIPH‐negative breast cancer (*P* = 0.0273, Figure 1D).

### 3.2 LIPH knockdown reduces the proportion of CD44^+^/CD24^−^ stem‐like cells

**FIGURE 2:**
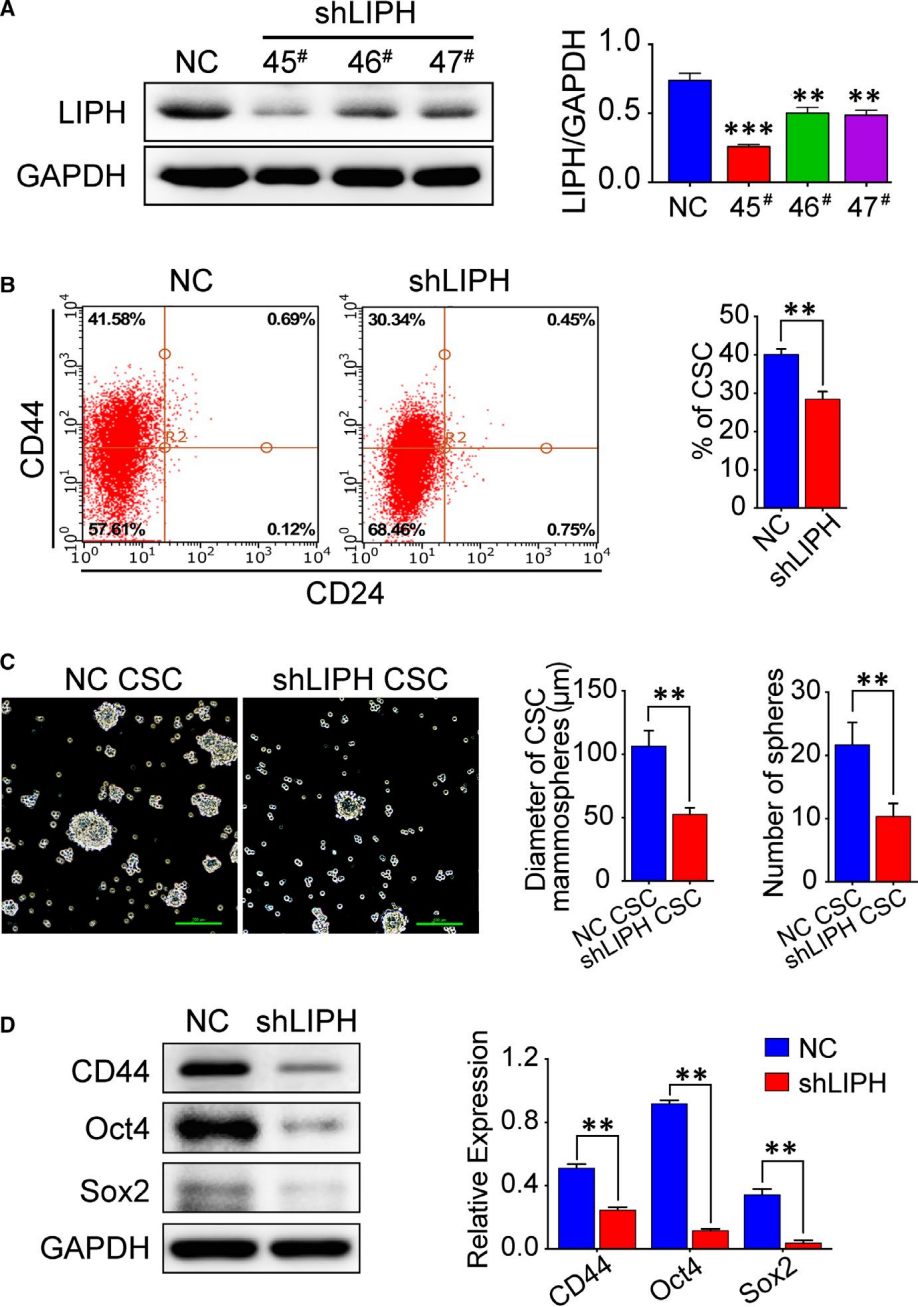
LIPH knockdown reduces the proportion of CD44^+^/CD24^−^ stem‐like cells. A, The relative levels of LIPH expression in breast cancer cells and shRNA‐transfected MDA‐MB‐231 cells. B, MDA‐MB‐231/shLIPH and MDA‐MB‐231/NC cells were double‐stained with CD24‐FITC and CD44‐PE antibody. Flow cytometry was used to analyse the population of CD44^+^/CD24^−^ cells. Representative images and quantitative results are shown. C, LIPH silencing decreased mammosphere formation and cell viability. Phase‐contrast images of MDA‐MB‐231 cells with LIPH knockdown. Scale bar, 100 µm. D, Western Blot analysis indicates the protein expression of the cancer stem cell markers, CD44, Sox2, and Oct4 in shLIPH and NC cells. Quantitative results are shown; *P < 0.05. Error bar, mean ± SEM

Given the findings from the clinical samples, we hypothesized that LIPH expression would increase the cancer stem‐like cell population. We analysed the CD44^+^/CD24^−^ cell population in shLIPH and NC cells by flow cytometry. We first knocked down LIPH in the MDA‐MB‐231 cells using a lentivirus transfection system (Figure 2A). The relative levels of LIPH expression were determined by Western Blot, which confirmed the efficacy of LIPH silencing, as LIPH expression was significantly lower in MDA‐MB‐231/shLIPH than in MDA‐MB‐231/NC cells (Figure 2A; *P* < 0.001). LIPH knockdown significantly decreased the stem‐like cancer cell population from 41.58% to 30.34% (*P* = 0.001, Figure 2B). In addition, LIPH knockdown reduced the diameter and number of cancer stem‐like cell mammospheres (Figure 2C). Moreover, down‐regulation of stem‐like cell markers, such as CD44, OCT4, and SOX2, upon LIPH silencing was detected by Western Blot in MDA‐MB‐231 cells (*P* < 0.001, Figure 2D). Thus, LIPH silencing decreased the population of breast cancer stem‐like cells.

### 3.3 LIPH knockdown mitigated the oxidation‐related OCR in the mitochondria and decelerated the metabolism of MDA‐MB‐231 cells

**FIGURE 3:**
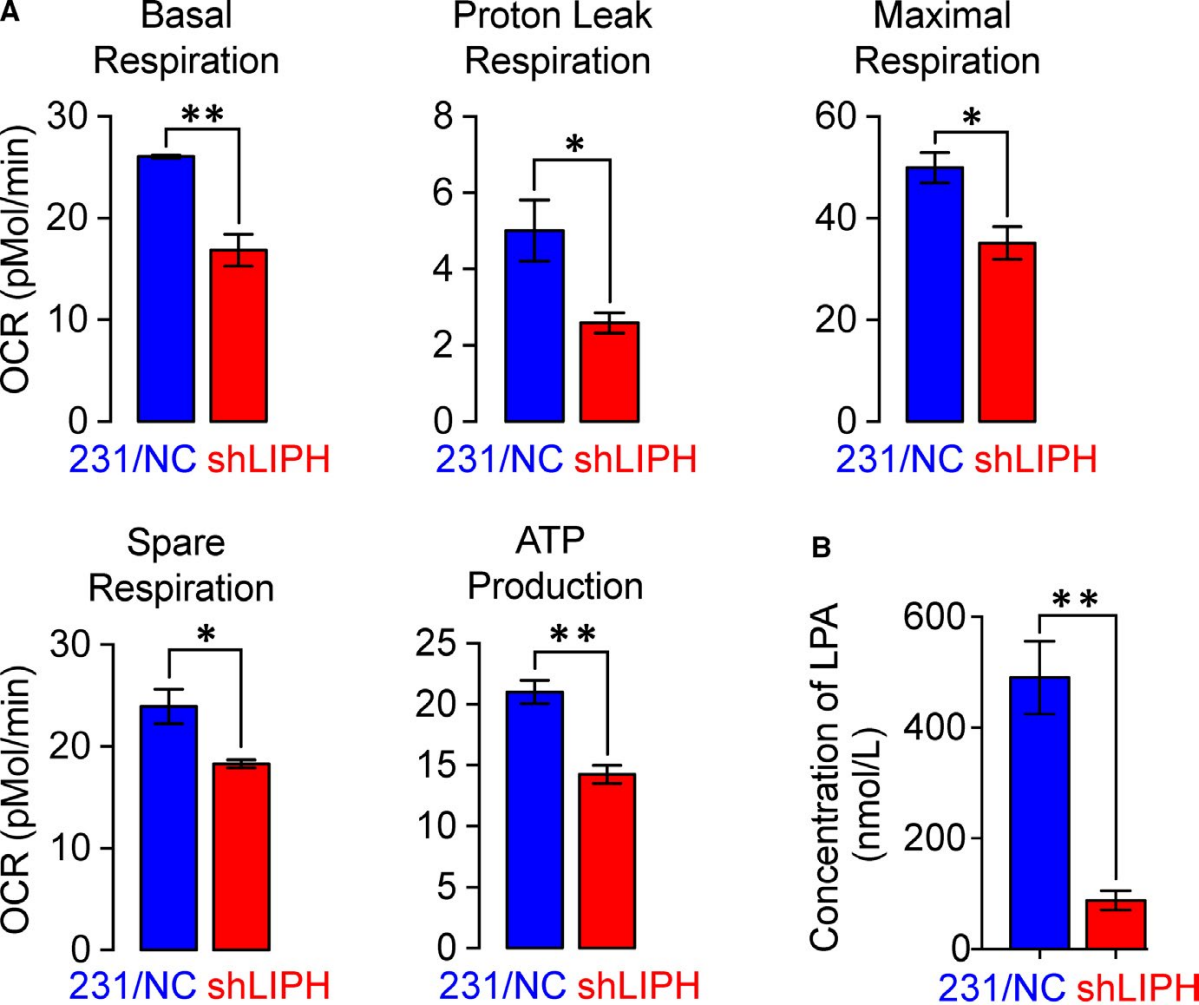
LIPH knockdown mitigates oxidation‐related OCR in the mitochondria and decelerated lysophosphatidic acid metabolism in MDA‐MB‐231 cells. A, Seahorse analysis of mitochondrial oxygen consumption rates (OCR) in MDA‐MB‐231/shLIPH and MDA‐MB‐231/NC cells. B, Comparison of lysophosphatidic acid (LPA) levels in MDA‐MB‐231/shLIPH and MDA‐MB‐231/NC cell culture supernatants. Data are expressed as mean ± SEM values from three independent experiments. *P < 0.05, **P < 0.01 vs control

We simultaneously measured changes in the OCR of control and LIPH‐silenced MDA‐MB‐231 cells using the XF24 extracellular flux analyser. LIPH silencing reduced the basal, ATP‐independent (proton leaking), maximal respiration, spare respiration, and ATP production (Figure 3A). In addition, LIPH silencing was significantly decreased by 2‐acyllysophosphatidic acid (LPA) production in MDA‐MB‐231 cells (*P* < 0.01, Figure 3B).

### 3.4 LIPH knockdown promotes apoptosis, arrests the cell cycle in G2/M phase, and inhibits invasion and migration in MDA‐MB‐231 cells

**FIGURE 4:**
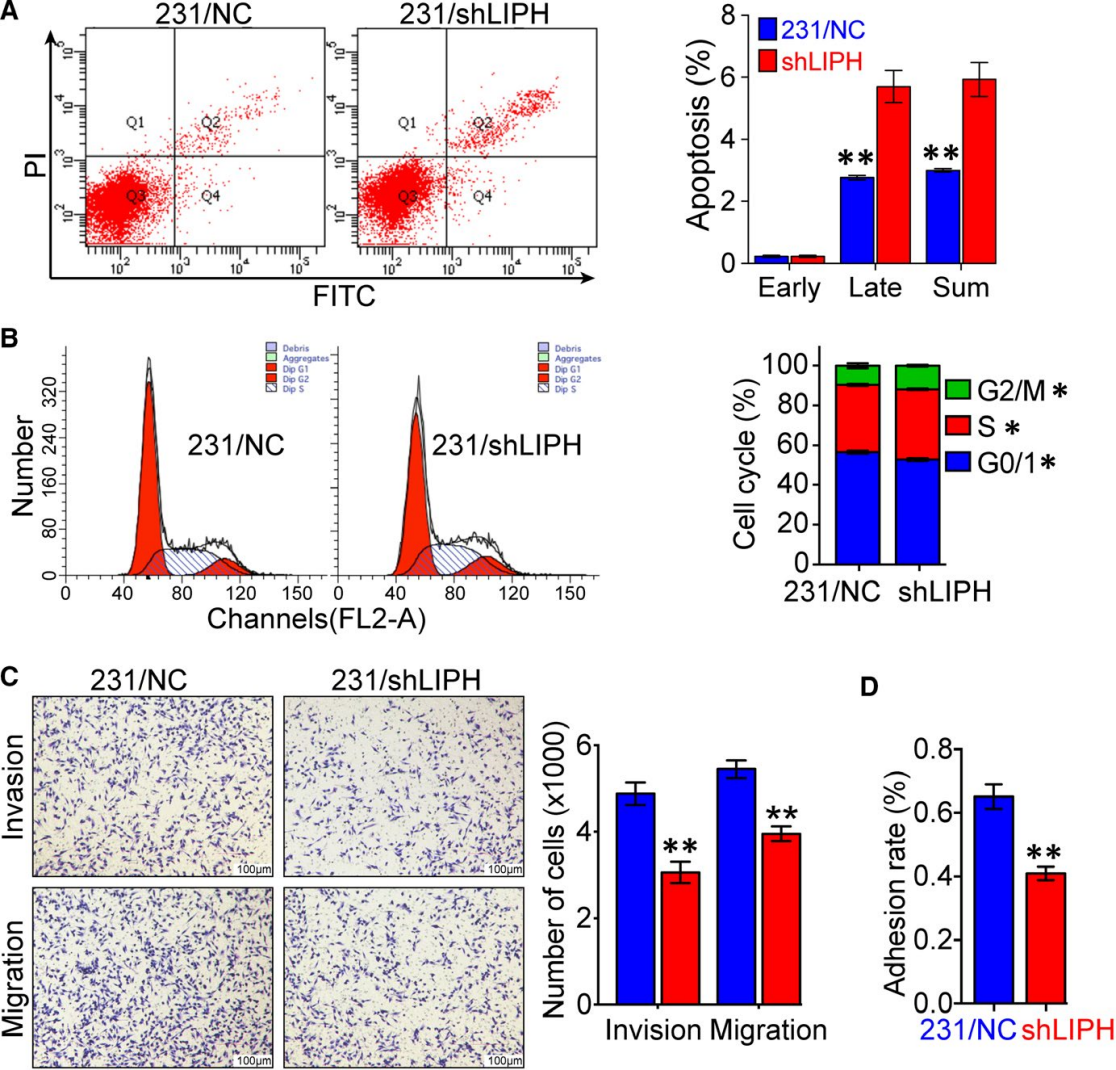
LIPH knockdown promotes apoptosis, arrests the cell cycle in the G2/M phase, and inhibits invasion and migration of MDA‐MB‐231 cells. A, Apoptosis in MDA‐MB‐231 cells, assessed by Annexin V‐FITC/PI staining assay. B, MDA‐MB‐231/shLIPH induces a G2/M cell cycle arrest in breast cancer cells, as shown by flow cytometry analysis. The effect of LIPH knockdown on the cell cycle in MDA‐MB‐231 cells is shown. C, Migration (lower) and invasion (upper) of shLIPH and control NC cells were (D) Comparison of the adhesion process in shLIPH and NC cells

About the effect of LIPH silencing on cell apoptosis, cell cycle, migration, and invasion of MDA‐MB‐231 cells, we found that LIPH silencing significantly increased the percentage of apoptotic cells from 3.0% to 6.9% (*P* = 0.006, Figure 4A) and induced G2/M arrest in MDA‐MB‐231 cells (*P* = 0.003, Figure 4B). Furthermore, LIPH silencing significantly decreased the number of migratory and invasive of MDA‐MB‐231 cells (*P* < 0.005 and 0.007, respectively, Figure 4C). Moreover, MDA‐MB‐231/shLIPH cells exhibited a significantly reduced adhesion capability (*P* < 0.001, Figure 4D).

### 3.5 LIPH has different biological functions, including integrin binding

**FIGURE 5:**
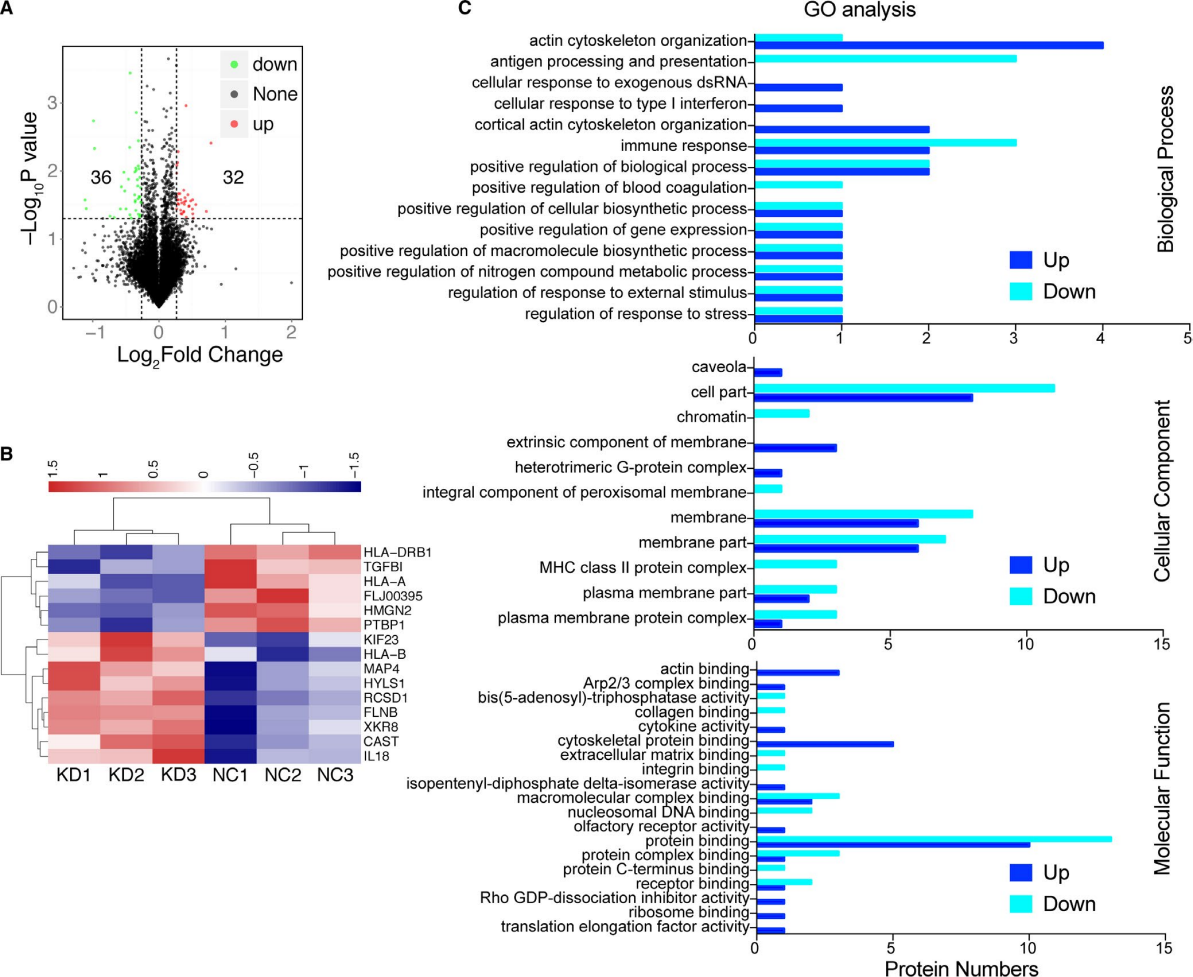
LIPH has various biological functions, including integrin binding. Total proteins were extracted from shLIPH and NC cells and labelled in triplicate with TMT10 tags. A, The differentially expressed proteins are indicated. B, A heat map illustrating the protein differential patterns (LIPH silencing‐up‐regulated and silencing‐down‐regulated proteins) is shown. C, The potential biological functions of differentially expressed proteins were analysed by GO biological process (upper), cellular component (medium), and molecular function (lower) annotations. The distribution of differentially expressed proteins is shown

To understand the mechanisms underlying the effects of LIPH silencing, protein expression profiles were determined in MDA‐MB‐231/NC and MDA‐MB‐231/shLIPH cells by tandem mass spectrometry (MS). Notably, 32 proteins were up‐regulated and 36 were down‐regulated in MDA‐MB‐231/shLIPH cells compared to control cells (Figure 5A). The 15 expression changes included 9 up‐regulated (KIF23, HLA‐B, MAP4, HYLS1, RCSD1, FLNB, XKR8, CAST, and IL18) and 6 down‐regulated (HLA‐DRB1, TGFB1, HLA‐A, FLJ00395, HMGN2, and PTBP1) proteins. GO biological process (BP) analysis indicated that the differentially expressed proteins were mainly involved in actin cytoskeleton organization and immune response, in addition to other processes (Figure 5B, upper part). Cellular component (CC) analysis assigned these proteins to cell part, membrane, and membrane part (Figure 5C, medium part), whereas molecular function (MF) annotation revealed their involvement in integrin binding, protein binding, and cytoskeletal protein binding functions (Figure 5C, bottom part). Thus, LIPH silencing substantially altered the protein expression profile in MDA‐MB‐231 cells.

**FIGURE 6:**
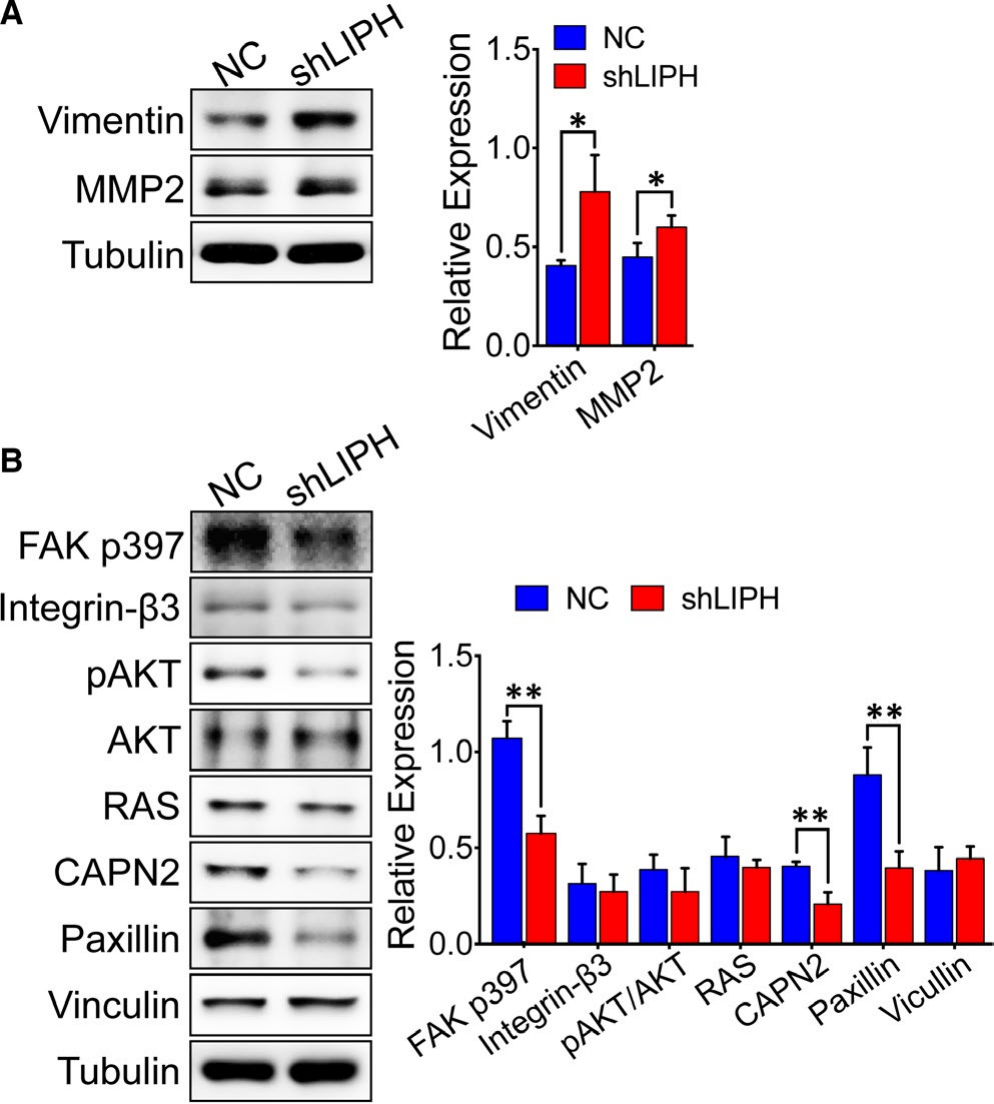
LIPH inhibits adhesion between tumour cells and enhances EMT. A, The relative adhesion activity between tumour cells related to tubulin protein expression in shLIPH and NC cells was determined by Western blot and quantified. B, The relative level of the mesenchymal‐epithelial transition was determined in shLIPH and NC cells by Western blot and quantified. Representative images are shown. Data are expressed as the mean ± SEM from three separate experiments. *P < 0.05, **P < 0.01 vs the control

We further employed Western blot analysis to verify whether LIPH silencing could affect the adhesion between tumour cells. As expected, MDA‐MB‐231/shLIPH cells exhibited a significant increase in adhesion markers compared to control cells, including vimentin and FAK p397 (*P* = 0.025 and 0.004, respectively, Figure 5A). Moreover, the relative levels of paxillin were significantly decreased in MDA‐MB‐231/shLIPH cells (*P* = 0.007, Figure 5B). These results were consistent with LIPH‐induced inhibition of cell‐cell adhesion. The level of CAPN2 was significantly decreased in MDA‐MB‐231/shLIPH cells (*P* = 0.007), whereas there was no significant difference in the relative levels of Integrin‐β, pAKT/AKT, RAS, and vinculin (*P* = 0.635, 0.237, 0.917, and 0.475, respectively, Figure 6B). Collectively, these data indicate that LIPH silencing attenuated adhesion between tumour cells and suppressed the EMT process in MDA‐MB‐231 cells.

## 4 DISCUSSION

Although the involvement of LIPH in breast cancer cell proliferation has been previously proposed, its exact pathogenetic role remains largely unknown.^16^ A recent study has shown that high expression of LIPG, another triglyceride lipase, is involved in TNBC metastasis through regulation of asymmetric cell division and maintenance of CSCs.^16^ Moreover, down‐regulation of LIPC in HepG2 cells is associated with decreased expression of CD133, reduced cell proliferation and colony formation, as well as increased chemotherapy resistance.^17^ CSCs are mainly responsible for the metastatic property of malignant cancers,^18^ raising our interest in whether LIPH is involved in the behavioural regulation of CSCs. We investigated whether LIPH plays an important role in the maintenance of CSCs and promotes metastasis in breast cancer cells. Interestingly, we found a high frequency of CD44^+^/CD24^−^ BCSCs in LIPH + TNBC tumours, where LIPH silencing decreased the ratio of CD44^+^/CD24^−^ stem‐like cells in vitro and blocked their mammosphere‐forming ability. In addition, the expression levels of cancer stem cell markers were decreased after LIPH knockdown. These findings indicate that LIPH expression promotes stemness in TNBC.

CSCs are associated with cell cycle regulation, metabolism, cell‐cell adhesion, and EMT.^18^ Following the frequency decrease of CD44^+^/CD24^−^ BCSCs, LIPH silencing promoted apoptosis, arrested the cell cycle in the G2/M phase, mitigated the oxidation‐related oxygen consumption rate in the mitochondria, and reduced metabolism. Finally, LIPH inhibited cell‐cell adhesion and enhanced EMT in our study. Thus, LIPH expression stimulates breast cancer cell mobility and may be crucial for metastasis.

As LIPH silencing reduced mitochondrial OCR and ATP production, it is likely to promote lipase‐independent functions through dysregulated mitochondrial biogenesis.^19^ Furthermore, LIPH silencing decreased lysophosphatidic acid (LPA) production in MDA‐MB‐231 cell supernatants in this study. LIPH specifically hydrolyses phosphatidic acid, producing 2‐acyl lysophosphatidic acid (LPA),^6^, ^20^ which has growth factor‐like biological activities.^21^ LPA is a ubiquitous lipid mediator that modulates cell migration and is implicated in tumour progression.^22^, ^23^ Thus, LIPH lipase‐dependent functions are required for LIPH‐induced enhancement of invasiveness, stemness, and basal/EMT features of breast cancer cells.

Tandem mass spectrometry shows that IPH silencing caused differential expression of 68 proteins belonging to different functional categories, including integrin binding, and was involved in various processes, such as actin cytoskeleton organization. We found that LIPH silencing significantly attenuated adhesion between tumour cells by modulating the FAK p397‐paxillin signalling axis in MDA‐MB‐231 cells. LIPH suppression increased the expression of FAK p397 and paxillin, two key factors in adhesion between tumour cells, suggesting that LIPH inhibits the adhesion between tumour cells and promotes EMT through CAPN2 regulation. Hence, our findings provide new insights into the molecular regulation of the LIPH/FAK p397/paxillin axis and TNBC progression.

**FIGURE 7:**
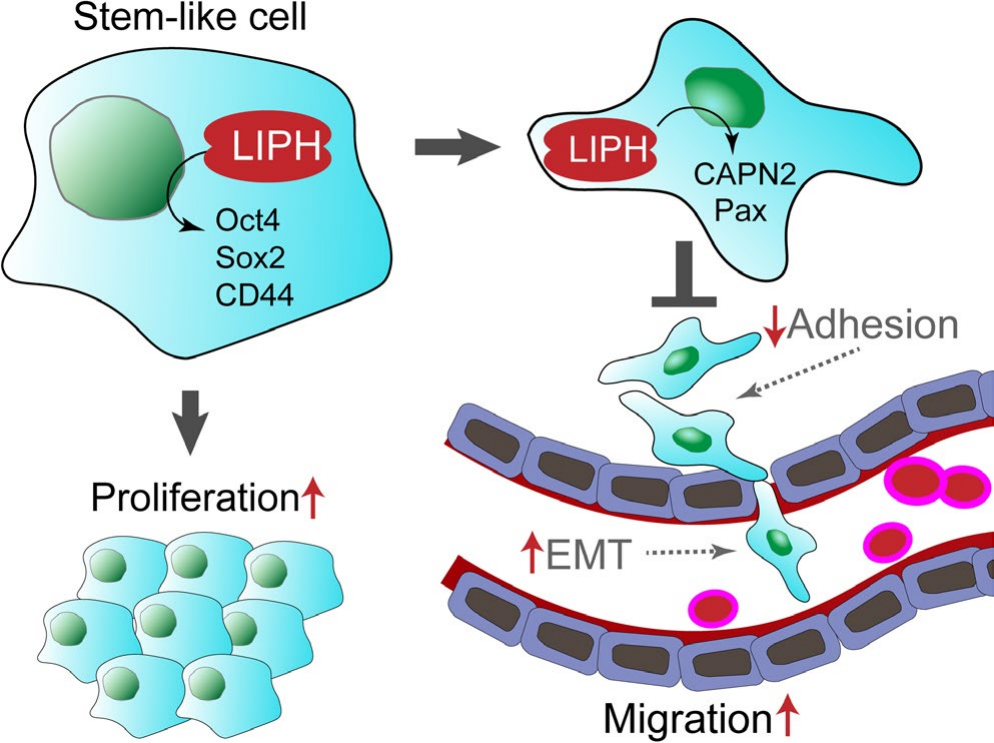
The schematic diagram of LIPH regulated metastasis in TNBC. The schematic diagram shows LIPH silencing reduced the ratio of stem‐like cells, induced the mesenchymal‐to‐epithelial transition, and inhibited metastasis, accounting for the increased aggressiveness of human LIPH‐positive TNBC

In short, our data suggest that LIPH is expressed in a portion of TNBC cells. LIPH expression was associated with lymph node and distant metastasis and predicted a disease‐free survival rate that was worse in patients with TNBC. LIPH silencing decreased the stem‐like cancer cell population, down‐regulating stem‐like cell markers (Sox2 and Oct‐4) in TNBC cells. As a result, the reduction in CD44^+^/CD24^−^ cells inhibited the proliferation, migration, and invasion of MDA‐MB‐231 cells, while mitigating oxidation‐related OCR in the mitochondria and decelerated the metabolism of MDA‐MB‐231 cells. LIPH can activate both CAPN2 and paxillin signalling pathways to regulate adhesion and metastasis (Figure 7). This study provides a strong foundation for the rational development of cancer immunotherapies targeting LIPH.

## CONFLICT OF INTEREST

These authors have declared that they do not have any competing interests.

## AUTHOR CONTRIBUTION

**Yixiao Zhang:** Conceptualization (supporting); Investigation (equal). **Xudong Zhu:** Investigation (supporting). **Xinbo Qiao:** Data curation (equal). **Xi Gu:** Data curation (equal). **Jinqi Xue:** Data curation (supporting). **Yanshuo Han:** Investigation (supporting); Writing‐original draft (supporting). **Lisha Sun:** Investigation (equal). **Meizi Cui:** Data curation (equal). **Caigang Liu:** Conceptualization (lead); Writing‐original draft (lead).

## Data Availability

These data can be available from the corresponding author.
