# Correction to “LIPH Promotes Metastasis by Enriching Stem‐Like Cells in Triple Negative Breast Cancer”

**DOI:** 10.1111/jcmm.70587

**Published:** 2025-05-19

Y. Zhang, X. Zhu, X. Qiao, et al., “LIPH Promotes Metastasis by Enriching Stem‐Like Cells in Triple Negative Breast Cancer,” *Journal of Cellular and Molecular Medicine* 24 (2020): 9125–9134.

In the published article, an error was identified in Figure 2, Panel B. The corrected figure is provided below. This revision does not impact the main findings or conclusions of the study. We sincerely apologise for the oversight.

**FIGURE 2:**
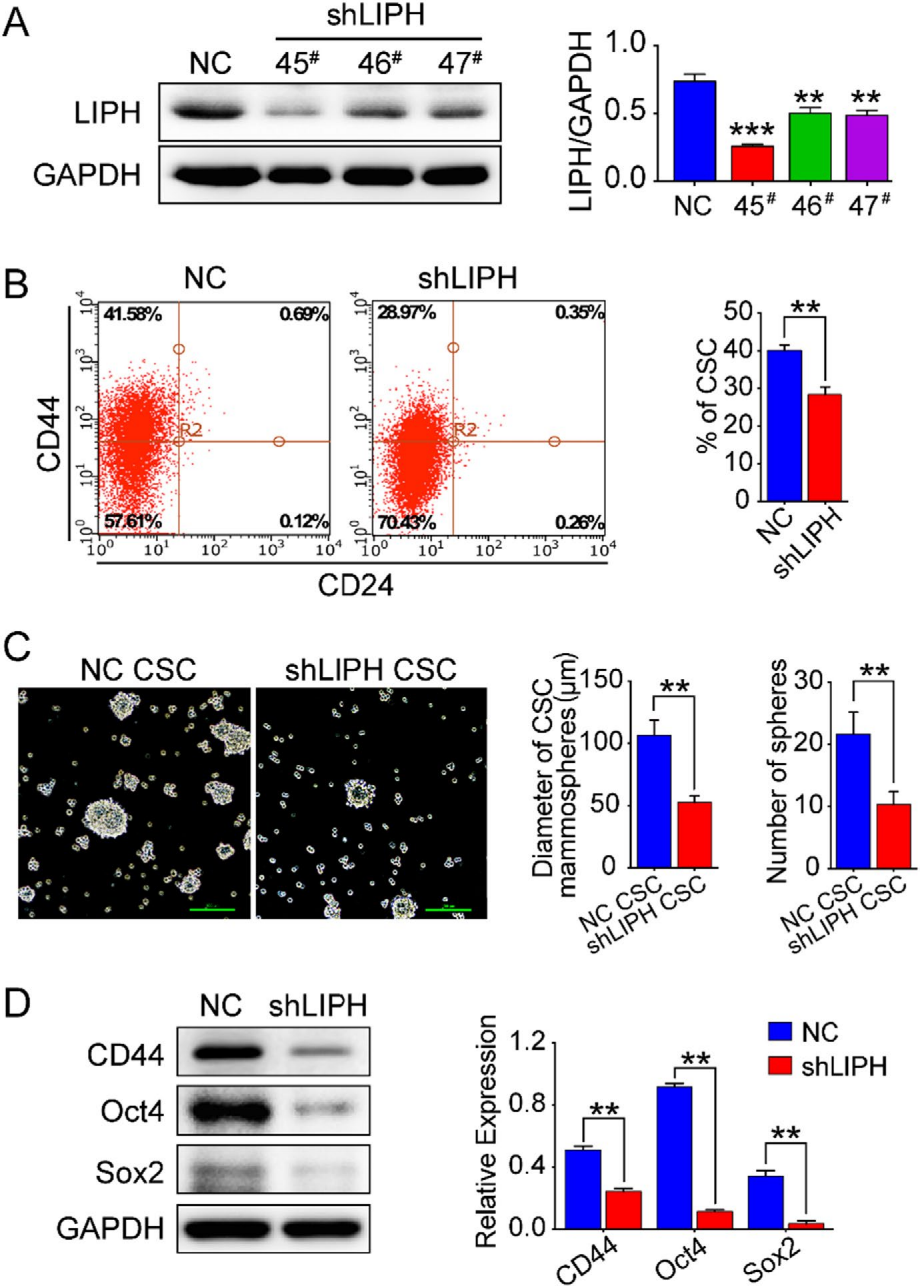
LIPH knockdown reduces the proportion of CD44^+^/CD24^−^ stem‐like cells. (A) The relative levels of LIPH expression in breast cancer cells and shRNA‐transfected MDA‐MB‐231 cells. (B) MDA‐MB‐231/shLIPH and MDA‐MB‐231/NC cells were double‐stained with CD24‐FITC and CD44‐PE antibody. Flow cytometry was used to analyse the population of CD44^+^/CD24^−^ cells. Representative images and quantitative results are shown. (C) LIPH silencing decreased mammosphere formation and cell viability. Phase‐contrast images of MDA‐MB‐231 cells with LIPH knockdown. Scale bar, 100 μm. (D) Western Blot analysis indicates the protein expression of the cancer stem cell markers, CD44, Sox2, and Oct4 in shLIPH and NC cells. Quantitative results are shown; *p < 0.05. Error bar, mean ± SEM.

